# Diagnosis and treatment of the Nutcracker syndrome: a review of the last 10 years

**DOI:** 10.1590/1677-5449.012417

**Published:** 2018

**Authors:** Guilherme Lourenço de Macedo, Matheus Alves dos Santos, Andrey Biff Sarris, Ricardo Zanetti Gomes

**Affiliations:** 1 Universidade Estadual de Ponta Grossa – UEPG, Departamento de Medicina Ponta Grossa, PR, Brasil.

**Keywords:** renal nutcracker syndrome, hematuria, nephrectomy, stents

## Abstract

The nutcracker syndrome is characterized by a group of clinical manifestations caused by compression of the Left Renal Vein. The main symptoms are: macro and micro hematuria, proteinuria, and flank pain. Diagnosis is usually made after excluding other causes, because there are no clinical criteria for diagnosis. Confirmation is by Doppler ultrasonography or computed tomography. Treatment can vary, depending on patient characteristics and the severity of the symptoms, while conservative treatment, open surgery, and endovascular surgery may be employed. Currently, open surgery is still the first-line treatment, but some less invasive approaches are gaining acceptance.

## INTRODUCTION

 The nutcracker syndrome is a rare clinical entity caused by compression of the left renal vein (LRV) by the superior mesenteric artery (SMA) as it passes between the SMA and the abdominal aorta (anterior nutcracker syndrome). 1 ^,^ 2 However, certain atypical variations of this arrangement have been described in the literature, the most common of which is posterior nutcracker syndrome, which occurs when the LRV is retroaortic and is subjected to compression between the spinal column and the abdominal aorta. 2 ^,^ 3 The compressive process causes varying levels of extrinsic stenosis of the renal branch, with results ranging from asymptomatic cases – in the majority of cases – to episodes of macroscopic hematuria, proteinuria, renovascular hypertension, flank pain, dyspareunia, dysmenorrhea, and pelvic varicose veins. 1 ^,^ 4 Other, rarer, manifestations include syncope, hypotension, and tachycardia (symptoms of autonomic dysfunction), Henoch-Schönlein purpura, Berger’s disease, membranous nephropathy, hypercalciuria, and nephrolithiasis. 5 ^,^ 6 The etiopathogenesis of pelvic pain is complex and hormones appear to play a contributing role, since female patients have more pain episodes and greater intensity of pain, especially during the premenstrual period, probably induced by progesterone levels. 4

 Certain reservations with regard to use of the terms “phenomenon” and “syndrome” should be noted. Although they are often treated as synonyms in the literature, “syndrome” should be reserved for cases in which the patient manifests symptoms, while “phenomenon” should be employed to refer to asymptomatic cases. 3

 While the syndrome apparently affects a higher proportion of female patients, its exact prevalence remains unknown. This is not simply due to the condition’s rarity, but also to the wide variability of its symptomatic presentations. Reported cases involve patients with ages ranging from infancy to the seventh decade of life, with greater prevalence among young adults (20 to 30 years) and middle-aged adults. 7 ^,^ 8

 Diagnosis is challenging and is generally made after exclusion of other more common causes, since there are no specific clinical diagnostic criteria. 9 Confirmation of the syndrome is by imaging exams, of which Doppler ultrasonography is the most widely used method. 1 ^,^ 2 Treatment varies depending on the severity of symptoms, ranging from conservative management for young patients or those with mild symptoms, to surgical and endovascular approaches for those who do not improve after conservative conduct or who have severe symptoms. 5

## METHOD

**Figure 1:**
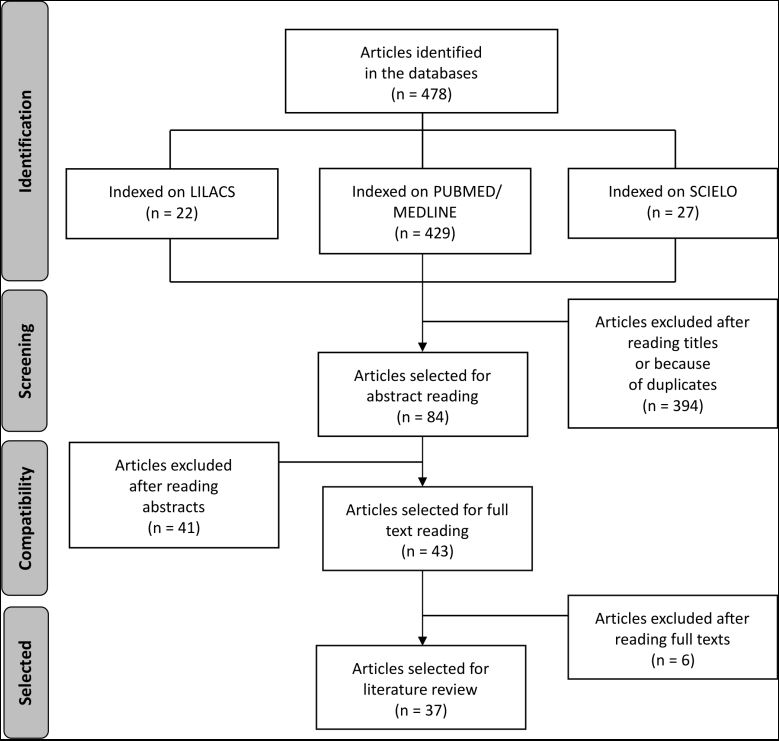
Search strategy for review of nutcracker syndrome.

 For this review, the LILACS, MEDLINE, PubMed, and SciELO databases were used to identify publications from 2007 to 2017 in English, Spanish, and Portuguese. Search expressions were constructed using the terms Renal Nutcracker Syndrome, Stents, Hematuria, and Nephrectomy and combinations of them with the Boolean operator AND. A total of 84 articles published during the preestablished period and covering the subject were selected for the study, after exclusion of items that were in other languages or were published before 2007. After analytical reading of the article abstracts, 37 articles were considered directly related to diagnosis and treatment, and were then read in full for analysis of criteria ( Figure 1 ). 

## COMMENTS

### Diagnosis

 Presence of the clinical presentations described above, especially recurrent and isolated macroscopic hematuria, when present in young patients, should arouse a diagnostic suspicion. 1 ^,^ 4 Although there are minimal findings reported in the literature, some studies have demonstrated a considerable number of cases in asymptomatic patients. Poyraz et al. 10 assessed 1,000 abdominal computed tomography scans with contrast and observed a 4.1% rate of diagnosis of nutcracker phenomenon. In the same study, just 8.8% of the patients diagnosed with LRV compression exhibited micro-hematuria or proteinuria with no other known etiology, and 5.5% of the patients had signs of varicocele or pelvic congestion. 10 Diagnosis of nutcracker syndrome is confirmed by imaging exams, as already mentioned. Venography with measurement of the renal vein pressure gradient is the gold-standard method, but its invasive nature makes it a later resort that is very often unnecessary for diagnosis. The initial investigative examination most widely used in clinical practice is Doppler ultrasound of the renal veins. In addition to enabling evaluation of flow, Doppler can also reveal the compressive process caused by the SMA. 1 ^,^ 2 Diagnostic criteria for the nutcracker syndrome include:

- A venous pressure gradient between the LRV and the inferior vena cava (IVC) ≥ 3 mmHg;
- A five times increase in maximum flow velocity in the LRV as it passes the SMA, in relation to the renal hilum;
- Computed tomography angiography or magnetic resonance angiography showing an angle between the aorta and the SMA of less than 45º. 1

The five times increase in flow velocity offers sensitivity of 80% and specificity close to 95% for nutcracker syndrome. 11 Doppler spectral analysis can also be used to measure the post-stenotic peak velocity, which normally exceeds 100 cm/s. 12

 Computed tomography and magnetic resonance imaging (MRI) are additional methods, but in the past they were little used for diagnosis of nutcracker syndrome. 1 However, recent studies have recommended tomography as the first diagnostic option because of its better accuracy and the opportunity it offers to conduct a wider assessment of abdominal findings. 2 ^,^ 10 Both tomography and MRI can show collateral circulation in the renal hilum, premature opacification of the left gonadal vein (LGV) (suggestive of reflux) and reduction of the aortomesenteric angle (< 10º). 12 The decision on whether to use tomography or Doppler ultrasound to investigate nutcracker syndrome should therefore be made on the basis of each patient’s characteristics: the urgency of diagnosis, exposure to radiation, cost and accessibility of the examination, other non-vascular abdominal diagnostic possibilities, and others. 

### Treatment

**Figure 2:**
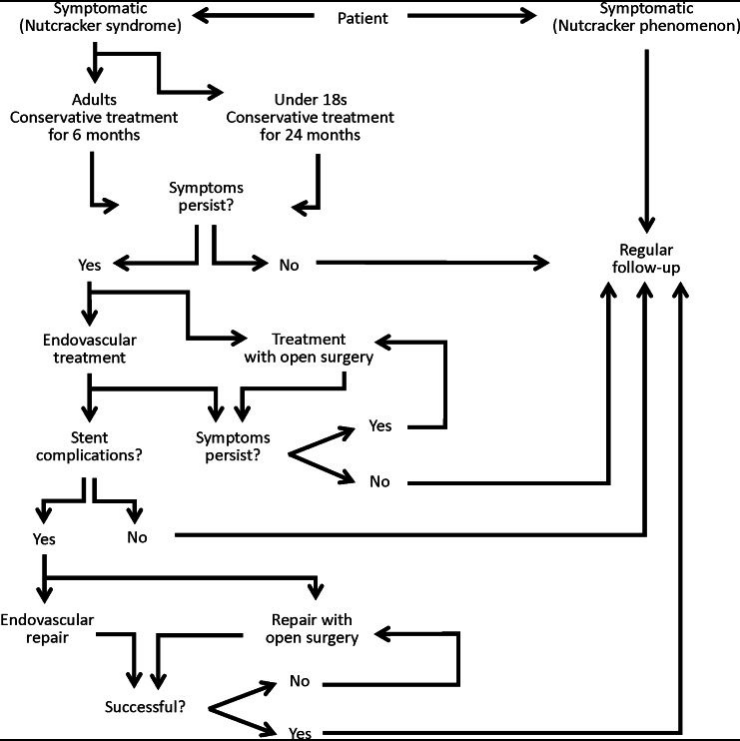
Treatment approach for nutcracker syndrome.

 Nutcracker syndrome is a disease with variable severity and symptomatic presentation that reflect the degree of LRV compression, renocaval hypertension, and development of collateral circulation. 5 Treatment of the syndrome is still a controversial subject, both with relation to choice of the best methods to be used for each patient and to the indications for treatment according to the diagnostic criteria employed. 7 ^,^ 8 Options vary from conservative treatment to nephrectomy, with countless invasive and endovascular surgical procedures between these two extremes. 3 The choice of treatment depends on the severity of symptoms, and interventions are generally reserved for patients who are symptomatic ( Figure 2 ). Procedures are guided by the expectations for reversal of the symptoms, by the stage of progression of the syndrome, and by the age of the patient, aiming not only to reduce hypertension in the LRV, but also to alleviate pelvic reflux in patients with venous congestion. 3 ^,^ 5

### Conservative treatment

 Conservative treatment is recommended for patients with discrete hematuria and mild symptoms. 5 ^,^ 7 ^,^ 8 Conservative treatment should be maintained for young patients, under the age of 18 years, for 24 months. 4 ^,^ 5 ^,^ 8 This is because physical development, growth of connective and adipose tissue close to the origin of the SMA and between it and the LRV, together with formation of collateral veins, may alleviate compression and venous hypertension, resulting in spontaneous remission from symptoms. 3 ^,^ 7 Around 75% of young patients with hematuria exhibited complete resolution of symptoms over this period. 4 ^,^ 5 ^,^ 7 ^,^ 8

 Adults should be monitored for at least 6 months before they are subjected to any procedures. This period is often sufficient for resolution of the condition in elderly patients with atypical and tolerable symptoms. 3 Low dose aspirin can be used to improve renal perfusion, although its use as routine has been questioned. 3 ^,^ 7 Additionally, angiotensin-converting enzyme inhibitors can help to relieve orthostatic proteinuria. 3 ^,^ 4 ^,^ 7 ^,^ 8 Elastic compression stockings can also be useful for patients with pelvic and flank pain. 13 ^,^ 14

### Surgical treatment

 The earliest descriptions of surgical interventions for nutcracker syndrome were published in the 1970s. Since then, a wide variety of techniques have been described. 13 ^,^ 14 Treatment is indicated in patients with severe symptoms, such as intense hematuria, combined or not with anemia, intense pelvic pain and pain in the abdominal flank, or symptoms persisting for more than 6 months in adults and 24 months in those less than 18 years old. 3 ^,^ 5 ^,^ 15 ^,^ 16 Surgical treatment is necessary to avoid development of chronic glomerulopathy and compromised renal function, permanent dilatation of the gonadal vein, and thrombosis of the renal vein. 5 ^,^ 15 Open surgery methods have been associated with greater morbidity, when compared to less invasive methods, because of the prolonged period of renal congestion, the need for additional anastomoses, and the extensive dissection involved. 5

#### Open surgery for anterior nutcracker syndrome

- Transposition of the LRV: this is the most common and effective surgery for treatment of anterior nutcracker syndrome and was first used for this purpose in 1982 by Stewart et al.. 3 ^,^ 17 It consists of sectioning the LRV and distal reimplantation to the IVC via a transabdominal, transperitoneal midline approach. 3 ^,^ 5 ^,^ 17 ^,^ 18 For patients who have an LRV with a permanent distortion caused by long term compression or those in whom the LRV is excessively tensioned after transposition, the great saphenous vein can be used as a patch or extension graft, respectively. 3 ^,^ 17 Despite the low postoperative risk, possible complications include deep venous thrombosis, retroperitoneal hematoma, paralytic ileus, and intestinal obstruction by adherences. 7 ^,^ 13 A considerable number of patients undergo restenosis and occlusion of the transposed vein and require reintervention. 19 The advantages of this operation are the short period of renal ischemia and few anastomoses, with high rates of symptomatic resolution, especially of the complaints of hematuria and flank pain, and it is considered the gold standard treatment for nutcracker syndrome. 7 ^,^ 14 ^,^ 18 Reed et al., conducted an 11-year study with 11 patients preferably treated with transposition of the LRV, observing resolution of hematuria in all patients and improvement or resolution of pain in eight of them, with no postoperative complications. They also reported a 27% reintervention rate. 20
- Left kidney autotransplantion: this is a highly invasive procedure consisting of nephrectomy and retransplantion of the kidney to the iliac fossa. 3 It is considered a complete procedure because it effectively normalizes LRV pressure levels and corrects any possible posterior renal ptosis, offers excellent results, and is associated with low morbidity. 3 ^,^ 7 ^,^ 21 However, there are additional risks that should be taken into consideration, such as the duration of renal ischemia, anastomoses of the renal artery and ureter, and the need for considerable surgical exposure. 3 ^,^ 7
- Transposition of the SMA: this procedure comprises transposition of the SMA from its origin at the aorta and reimplantation at a point below the LRV, which is a more complex procedure when compared to venous transposition, but is a surgical option that requires little retroperitoneal exposure and reduces the risk of LRV thrombosis. 3 ^,^ 7 ^,^ 16 However, it is rarely used because of the significant risk of arterial thrombosis – secondary to the reastomosis – and of mesenteric ischemia, combined with an elevated rate of postoperative complications. 3 ^,^ 7 ^,^ 16 ^,^ 18
- Nephropexy: a treatment method initially described in the 1980s. 16 Simple nephropexy with excision of varicosities, which is a procedure that only resolves renal ptosis, has been discouraged because it does not treat the primary pathophysiology. 21 ^,^ 22 According to Hmida et al., nephropexy is used as an etiologic treatment when it is conducted concurrently with lowering of the LRV, via lobectomy with a retroperitoneal approach. Since this procedure involves a low risk of injury to intraperitoneal organs, it can be considered a treatment option for young patients. 22
- Nephrectomy: this is the most radical of the surgical procedures and is recommended in cases in which hematuria persists after different therapeutic approaches, especially after transposition of the LRV. 7
- Renocaval bypass: this technique employs the great saphenous vein to construct a bypass and does not require transposition of the LRV. 17 The saphenous vein is anastomosed proximally to the IVC, below the LRV, and distally to the LRV. Both anastomoses are performed with partial clamping so that they have little effect on venous hemodynamics. There is no need to ligate the lumbar veins, the gonadal vein, or the left renal vein if they are not refluxing, since they do not affect the anastomoses. There are limited long-term results available and little experience with this procedure. 3
- Transposition of the LGV: in this procedure the LGV is exposed via the transverse mesocolon, isolated, transected distally and reimplanted to the IVC below the inferior mesenteric vessels with interrupted sutures using 6.0 polypropylene thread. 3 ^,^ 17

Laparoscopic techniques have been increasing in popularity as minimally invasive surgery techniques improve. 18 Laparoscopic nephrectomy and autotransplantion avoid extensive abdominal incisions and are associated with reduced postoperative morbidity and discomfort and shorter hospital stays. 16 Procedures for splenorenal bypass and LRV transposition by laparoscopy have also been described. Both techniques offer patients improvements to symptoms and pain; however, the splenorenal technique involves a risk of causing complications involving the spleen and LRV hypertension is unaffected. 7 ^,^ 18 In 2015, Thaveau et al. 23 described using robot systems for transposition of the LRV, followed by embolization of the ovarian vein, reporting that 6 months after the operation the patient was asymptomatic. 

#### Open surgery for posterior nutcracker syndrome

 A retroaortic LRV compressed between the spinal column and the abdominal aorta produces the same symptomology as the anterior variant of the syndrome. 17 Open surgery for anterior transposition of the LRV has become the treatment recommended for posterior nutcracker syndrome. 24 This procedure consists of excision of the LRV, leaving a small margin from the IVC wall, translocation of the vein to a position anterior of the aorta, and reimplantation to the IVC – generally in a position superior to its previous location, in order to relieve the pressure. 17 Deser et al. 25 report successful use of a polytetrafluoroethylene (PTFE) prosthetic graft for renocaval bypass in a patient with nutcracker. However, PTFE is not recommended as a first-line option for this procedure because of the risk of early thrombosis and infection. 3

### Endovascular treatment

 Endovascular surgery is a form of intervention that is becoming ever more popular among specialists for treatment of vascular lesions, including nutcracker syndrome. 13 ^,^ 14 ^,^ 26 ^,^ 27 Although many surgeons still choose traditional open surgery methods, this type of intervention involves a greater risk of morbidity and of complications, when compared with less invasive approaches. 13 Thus, since reported cases in which endovascular treatment has been used have achieved satisfactory results for treatment of venous obstruction diseases, these approaches are increasingly recommended by researchers. 26 One of the largest studies undertaken with this type of technique to date compared 15 patients treated using endovascular methods with 5 patients treated using open surgery. It showed that in the years following these procedures, all patients treated with stents were asymptomatic, although two had persistent microscopic hematuria after physical exercise and one stent migrated to the right atrium, requiring surgery. 28 ^,^ 29

 At least 150 successful cases of endovascular treatment have been reported in the medical literature. 27 However, information on long-term follow-up is still lacking, which justifies the reluctance to use it with young patients. 8 ^,^ 30 ^,^ 31 While it is a simple and attractive option, complications include stent migration, fractures, and venous occlusion. 28 The techniques used are embolization of the LGV and stent placement. 15

#### Stents

 Endovascular stent placement is an alternative treatment option. It is usually preferable to open surgery because of the long period of renal congestion, because of the greater likelihood of complications in these cases, and because of the need for extensive dissection in this type of operation. 6

 The first report of using vascular endoprostheses to treat nutcracker syndrome dates from the 1990s. 13 ^,^ 14 ^,^ 18 ^,^ 28 Wallstents® were used and these are still the devices most preferred by surgeons, although since then many other models have been used, with good results, such as the spiral Z-stent®, Nikki® stent, Palmaz® stent, and SmartControl® stent. 13 ^,^ 32 Two of the most important studies of this subject, both Chinese, confirm the success of endovascular methods. In 2011, Chen et al. reported follow-up of 61 patients for 5 years and 6 months, reporting excellent or good improvement for 59 patients, with total resolution or improvement of symptoms of flank pain, hematuria, and proteinuria. 32 In 2012, Wang et al. 26 analyzed 30 patients, in 29 of whom the renocaval pressure gradient was successfully reduced (calculated by comparison of measurements taken before and after procedures), with regression of complaints of pain, hematuria, and varicocele on the left side in male patients within 6 months of stent placement. Compression of the LRV is relieved by stenting in the majority of patients, although it is not known whether the drop in renocaval pressure occurs immediately or gradually. 32

 The ideal stent should have enough radial strength to eliminate stenosis, good conformability to fit the epithelium of the vessel and little length shrinkage to enable adequate positioning. 13 ^,^ 26 ^,^ 32 The SmartControl® stent combines high radial strength and flexibility with less than 8% shrinkage in length. The Palmaz® stent and the Wallstent® exhibit greater device contraction, which can reach from 5 to 25% and more than 30%, respectively. 32 Self-expanding stents (Wallstent®) are the most frequently employed, and the recommendation is that a device with a length of 6 or 8 centimeters should be used, positioned at the first division of the LRV. 26 Additionally, in order to avoid migration of the device, it is suggested that the stent should be around 20% larger than the venous diameter at the renal hilum. The basic size is considered to be 16 mm in diameter by 60 mm in length for patients with occidental ancestry and 14 mm in diameter by 60 mm in length for patients with oriental ancestry, because of the smaller anatomic proportions characteristics of the latter population. 32 Use of balloon catheter angioplasty is controversial and is not essential. 13 ^,^ 26 ^,^ 32 Chen et al. recommend that balloon expansion should only be used in cases in which stenosis of the LRV remains after stent placement. 32

 Patients must take anticoagulant medication and antiplatelet drugs for 2 to 3 months, which is the time needed for complete endothelization of the stent. 19 ^,^ 33 The therapeutic regimen recommended is 3 days on low molecular weight heparin, 30 days on clopidogrel, and 3 months on aspirin. 33

 The principal complications of this intervention method include incorrect stent placement, device migration, partial displacement of the stent into the IVC, and migration to the renal hilum region of the LRV. 7 Less common complications include embolization of the stent, in-stent restenosis, and thrombosis or fracture resulting in venous occlusion. 7 ^,^ 19 ^,^ 32 These complications are primarily due to type and size of the device, balloon dilation, and insufficient knowledge on the part of the surgeon who conducts the procedure. 7 ^,^ 34

 Even though the current literature suggests that the procedure is safe, caution is still needed, because the stent migration rate is 7.3% for all cases reported to date. The reasons for migration may be the effect of cardiac movements, activities too soon after surgery, incompatibility between the diameter of the LRV and the diameter of the stent, or incorrect positioning. 29 The first-line, and safest, option for treatment of stent migration is percutaneous removal. 32 However, under certain circumstances, such as migration to the heart, use of a special stent, or endothelization in an inappropriate location, percutaneous removal may be difficult or impossible, requiring surgical intervention, which is a procedure associated with high morbidity rates. 32 ^,^ 35

 One type of stent that is mentioned little in the literature is the extravascular device. Compared to endovascular stents, it is a less invasive option, if the possibility of device migration is taken into account. Intravascular stents should be recommended with caution, especially when the patient is a child or adolescent, since the lumen of the LRV can increase in diameter and the device may no longer serve its purpose as physical development progresses. The extravascular device is an alternative option for maintaining the device in the correct position. 29

#### Embolization of the LGV

 Once nutcracker syndrome has set in, one of its possible consequences is insufficient blood volume outflow from the LGV, causing a buildup of blood and dilatation of its walls, which can even lead to rupture. 8 Patients with this syndrome, with or without hematuria, may have symptoms of gonadal vein insufficiency, which manifest as varicocele in men and pelvic congestion syndrome in woman. Although treatment of the LRV stenosis can alleviate gonadal reflux, it is generally not effective for improving the symptoms. 3 In these cases, endovascular embolization of the gonadal veins can achieve relief from symptoms in 56 to 98% of patients. In rare cases, complications include coil displacement into the lung. 8

 Since its introduction in 1993, transcatheter embolization of the LGV has become the gold standard for treatment of pelvic congestion syndrome secondary to insufficiency of ovarian and pelvic veins. Several embolic agents have been described, including sclerosing foam, glue, vascular plugs, and coils. 36

 The procedure is typically conducted via a femoral access, although jugular access can also be used. 3 Imaging examinations of the LRV should be performed to confirm drainage of the left kidney, since patients with severe compression may be heavily dependent on pelvic flow. 3 ^,^ 37

 According to the current literature, the decision on whether to treat one or both ovarian veins should be taken on the basis of the severity of symptoms, the degree of reflux through each ovarian vein, and the anatomy of pelvic varicosities. Clinical analysis and experience should be used to direct embolization therapy in conjunction with symptoms, anatomy, and functional studies. 36

## CONCLUSIONS

 Although rare, nutcracker syndrome is present in medical practice. It is therefore necessary to learn about it to be in a position to correctly diagnose and manage patients. Clinical findings and a high degree of diagnostic suspicion are decisive factors for establishing the ideal conduct, especially in patients who have not undergone imaging exams previously. There is a wide range of surgical options and only profound theoretical and practical mastery of these techniques enables the correct choice to be made for the various different types of patients with the syndrome. It is therefore evident that there is a need to improve underlying theoretical knowledge of nutcracker syndrome, which is the objective of this review. 
