# Supplementary material for: Diagnosis and treatment of the Nutcracker syndrome: a review of the last 10 years
Source: J Vasc Bras. 2018 Jul-Sep;17(3):220–8. doi: 10.1590/1677-5449.012417 (PMC6326141; doi:10.1590/1677-5449.012417)
Supplement: Translation

# Translation: Diagnóstico e tratamento da síndrome de quebra-nozes (*nutcracker* ): revisão dos últimos 10 anos

**Authors:** Guilherme Lourenço de Macedo, Matheus Alves dos Santos, Andrey Biff Sarris, Ricardo Zanetti Gomes

**Affiliations:** 1 Universidade Estadual de Ponta Grossa – UEPG, Departamento de Medicina, Ponta Grossa, PR, Brasil.

**Keywords:** síndrome de quebra-nozes, hematúria, nefrectomia, stents

## Abstract

A síndrome de quebra-nozes é caracterizada por um grupo de manifestações clínicas que ocorrem por conta da compressão da veia renal esquerda. Seus principais sintomas são macro e micro-hematúria, proteinúria e dor no flanco. O diagnóstico é geralmente realizado após a exclusão de outras causas mais comuns, por conta da ausência de critérios clínicos para diagnóstico. Sua confirmação é feita por exames de imagem, com uso da ultrassonografia Doppler e tomografia computadorizada. O tratamento pode variar com as características do paciente e com a gravidade dos sintomas, e inclui o tratamento conservador, a cirurgia aberta e a cirurgia endovascular. Atualmente, a cirurgia aberta continua sendo a linha de frente, mas abordagens menos invasivas vêm ganhando cada vez mais espaço.

## INTRODUÇÃO

 A síndrome de quebra-nozes (SQN) (*nutcracker syndrome*), entidade clínica rara, consiste na compressão da veia renal esquerda (VRE) pela artéria mesentérica superior (AMS) em sua passagem entre esta e a aorta abdominal (SQN anterior) 1 ^,^ 2 . Algumas variações atípicas dessa disposição, no entanto, já foram descritas na literatura, entre as quais se destaca a SQN posterior, que ocorre quando a VRE é retroaórtica e passa a ser comprimida entre a coluna vertebral e a aorta abdominal 2 ^,^ 3 . O processo compressivo leva a diferentes níveis de estenose extrínseca do ramo renal, acarretando desde quadros assintomáticos – o que ocorre na maioria dos casos – até episódios de hematúria macroscópica, proteinúria, hipertensão renovascular, dor em flancos, dispareunia, dismenorreia e varizes pélvicas 1 ^,^ 4 . Síncope, hipotensão e taquicardia (sintomas de disfunção autonômica), púrpura de Henoch-Schönlein, doença de Berger, nefropatia membranosa, hipercalciúria e nefrolitíase são manifestações que podem raramente se associar 5 ^,^ 6 . A etiopatogenia da dor pélvica é complexa e o papel hormonal parece contribuir, já que pacientes do sexo feminino têm aumento da intensidade e dos episódios de dor especialmente no período pré-menstrual, induzido, provavelmente, pelos níveis de progesterona 4 . 

 Ressalvas quanto ao emprego dos termos “fenômeno” e “síndrome” devem ser feitas. Apesar de serem comumente utilizados como sinônimos na literatura, o uso de “síndrome” deve se limitar às ocasiões em que o paciente apresente sintomas. “Fenômeno”, por sua vez, deverá ser empregado em casos de pacientes assintomáticos 3 . 

 Embora a síndrome afete, aparentemente, um número superior de pacientes do sexo feminino, sua exata prevalência permanece desconhecida. Isso se deve não só à raridade com que ocorre, mas também à extensa variabilidade de apresentação sintomática. Os casos registrados envolvem pacientes com idades que variam desde o período da infância até a septuagésima década, com maior prevalência em pacientes jovens (entre 20 e 30 anos) e adultos de meia-idade 7 ^,^ 8 . 

 O diagnóstico é desafiador, geralmente feito após a exclusão de outras causas mais comuns, haja vista a ausência de critérios clínicos específicos de diagnóstico 9 . A confirmação da síndrome é feita por exames de imagem, sendo a ultrassonografia com Doppler o método mais utilizado 1 ^,^ 2 . O tratamento varia de acordo com a gravidade sintomática, abrangendo abordagens conservadoras para pacientes jovens ou com sintomas leves, ou abordagens cirúrgicas e endovasculares para aqueles que não obtiveram melhora após a terapia conservadora ou que apresentam sintomas severos 5 . 

## MÉTODO

 Para a presente revisão, foram consideradas publicações nas bases de dados LILACS, MEDLINE, PubMed e SciELO que estivessem compreendidas no período de 2007 a 2017, nos idiomas inglês, espanhol e português. Para a expressão da pesquisa, foram utilizados os descritores *Renal Nutcracker Syndrome, Stents, Hematuria* e *Nephrectomy,* bem como a combinação entre elas através do operador booleano AND. Selecionaram-se 84 artigos datados do período pré-estabelecido e que abordavam a temática deste estudo, excluindo-se aqueles que se apresentavam em outro idioma ou de épocas anteriores. Após leitura analítica com base nos resumos dos artigos, consideraram-se 37 trabalhos relacionados diretamente com diagnóstico e tratamento, que foram lidos na íntegra para análise dos critérios ( Figura 1 ). 

**Figura 1:** Estratégia de busca usada para revisão da literatura sobre a síndrome de quebra-nozes.

## COMENTÁRIOS

### Diagnóstico

 A presença clínica supracitada, especialmente hematúria macroscópica recorrente e isolada, quando presente em pacientes jovens, deve levar à suspeição diagnóstica 1 ^,^ 4 . Apesar de os achados da literatura serem mínimos, alguns estudos têm demonstrado um número importante de casos de pacientes assintomáticos. Poyraz et al. 10 avaliaram 1.000 tomografias computadorizadas de abdome com contraste e observaram 4,1% de diagnósticos do fenômeno de quebra-nozes. Ainda nesse estudo, apenas 8,8% dos pacientes diagnosticados com compressão da VRE apresentavam micro-hematúria ou proteinúria sem outra etiologia conhecida, e 5,5% dos pacientes ainda apresentavam sinais de varicocele ou congestão pélvica 10 . O diagnóstico confirmatório da SQN é realizado por exame de imagem, como já referido. A venografia com aferição de gradiente de pressão da veia renal é o método padrão-ouro, mas o caráter invasivo a deixa como opção tardia, muitas vezes desnecessária, para o diagnóstico. O exame de avaliação inicial mais utilizado na prática clínica é o ultrassom com Doppler de veias renais. Além de permitir a avaliação do fluxo, o Doppler possibilita a visualização do processo compressivo efetuado pela AMS 1 ^,^ 2 . Entre os critérios diagnósticos da SQN, têm-se:

- Existência de um gradiente venoso entre a VRE e a veia cava inferior (VCI) ≥ 3 mmHg;
- Aumento de cinco vezes na velocidade máxima do fluxo na VRE na passagem pela AMS em relação ao hilo renal;
- Angiotomografia ou angiorresonância com ângulo entre a aorta e a AMS inferior a 45º 1 .

O aumento de cinco vezes na velocidade de fluxo corresponde a uma sensibilidade de 80% e uma especificidade próxima de 95% para SQN 11 . Com o uso do Doppler espectral, ainda é possível medir a velocidade de pico pós-estenótica, que normalmente se encontra acima de 100 cm/s 12 . 

 A tomografia computadorizada e a ressonância magnética são métodos adicionais, porém pouco utilizados no passado, para o diagnóstico de SQN 1 . No entanto, novos estudos têm apontado para o uso da tomografia como primeira opção diagnóstica devido à sua maior acurácia, além de avaliação mais ampla dos achados abdominais 2 ^,^ 10 . A tomografia e a ressonância podem demonstrar circulação colateral no hilo renal, opacificação precoce da veia gonadal esquerda (VGE) (inferindo refluxo) e redução do ângulo aortomesentérico (< 10º) 12 . Sendo assim, a decisão do uso de tomografia ou Doppler para avaliação de SQN deve recair sobre as características de cada paciente – urgência do diagnóstico, exposição à radiação, custo e acessibilidade do exame, outras suspeitas diagnósticas abdominais não vasculares, entre outras. 

### Tratamento

 A SQN é uma doença de gravidade e apresentação sintomática variável que reflete o grau de compressão da VRE, de hipertensão renocaval e de desenvolvimento colateral de circulação 5 . O tratamento da síndrome ainda permanece um tópico controverso, tanto na escolha da melhor modalidade a ser utilizada para cada paciente quanto nas indicações para tratamento de acordo com os critérios diagnósticos utilizados 7 ^,^ 8 . As opções variam desde tratamento conservador até nefrectomia, com inúmeros procedimentos cirúrgicos invasivos e endovasculares entre esses dois extremos 3 . A escolha terapêutica depende da gravidade dos sintomas, e as intervenções estão reservadas, geralmente, aos pacientes que se encontrem sintomáticos ( Figura 2 ). Os procedimentos são guiados pela expectativa de reversão dos sintomas, pelo estágio de evolução da síndrome e pela idade do paciente, objetivando não só a redução da hipertensão na VRE, mas também a redução do refluxo pélvico em pacientes que apresentem congestão venosa 3 ^,^ 5 . 

**Figura 2:** Abordagem terapêutica da síndrome de quebra-nozes.

### Tratamento conservador

 É recomendado aos pacientes com hematúria discreta e pouco sintomáticos 5 ^,^ 7 ^,^ 8 . A abordagem conservadora deverá ser mantida em pacientes jovens, menores de 18 anos, durante 24 meses 4 ^,^ 5 ^,^ 8 . Isso porque o desenvolvimento físico, o crescimento de tecido conjuntivo e adiposo próximo à origem da AMS e entre esta e a VRE, juntamente à formação de veias colaterais, podem aliviar a compressão e a hipertensão venosa, resultando em remissão espontânea dos sintomas 3 ^,^ 7 . Cerca de 75% dos pacientes jovens com hematúria tiveram resolução completa dos sintomas nesse período 4 ^,^ 5 ^,^ 7 ^,^ 8 . 

 Em adultos, o acompanhamento deve ser de, ao menos, 6 meses antes da realização de qualquer procedimento. Frequentemente, esse período é suficiente para a resolução do quadro em pacientes idosos com sintomas atípicos e toleráveis 3 . Baixas doses de aspirina para melhorar a perfusão renal podem ser utilizadas, embora seu uso rotineiro seja motivo de questionamento 3 ^,^ 7 . Além disso, a utilização de inibidores da enzima conversora de angiotensina pode contribuir para o alívio da proteinúria ortostática 3 ^,^ 4 ^,^ 7 ^,^ 8 . O uso de meias elásticas de compressão para pacientes com dor pélvica e no flanco também pode ser útil 13 ^,^ 14 . 

### Tratamento cirúrgico

 As primeiras descrições de intervenções cirúrgicas para a SQN ocorreram na década de 1970. Desde então, uma ampla variedade de técnicas têm sido registradas 13 ^,^ 14 . As indicações terapêuticas são para pacientes que se apresentem com sintomas severos, como hematúria intensa, acompanhada ou não de anemia, dor pélvica e no flanco abdominal de forte intensidade, ou sintomas persistentes por período superior a 6 meses no adulto e 24 meses em menores de 18 anos 3 ^,^ 5 ^,^ 15 ^,^ 16 . O tratamento cirúrgico se faz necessário para evitar o desenvolvimento de glomerulopatia crônica e comprometimento da função renal, bem como a dilatação permanente da veia gonadal e trombose da veia renal 5 ^,^ 15 . Os métodos cirúrgicos abertos têm sido associados a uma maior morbidade, se comparados aos métodos menos invasivos, devido ao longo período de congestão renal, à necessidade de anastomoses adicionais e à extensa dissecção 5 . 

#### Cirurgia aberta para SQN anterior

- Transposição da VRE: é a cirurgia mais comum e efetiva para o tratamento de SQN anterior, aplicada primeiramente em 1982 por Stewart et al. 3 ^,^ 17 . Consiste na secção da VRE e reimplantação distal na VCI por meio de abordagem medial transabdominal transperitoneal 3 ^,^ 5 ^,^ 17 ^,^ 18 . Para pacientes que apresentarem VRE com distorção permanente devido ao longo tempo de compressão ou aqueles em que a VRE se encontrar muito tensionada após a transposição, a veia safena magna pode ser utilizada como remendo e enxerto de extensão, respectivamente 3 ^,^ 17 . Apesar do baixo risco pós-operatório, as complicações possíveis incluem trombose venosa profunda, hematoma retroperitoneal, íleo paralítico e obstrução intestinal por aderência 7 ^,^ 13 . Um número considerável de pacientes apresenta reestenose e oclusão da veia transposta, requerendo novas intervenções 19 . As vantagens dessa cirurgia são o curto período de isquemia renal e poucas anastomoses, com altas taxas de resolução sintomática, em especial das queixas de hematúria e dor no flanco, tornando-a o padrão-ouro no tratamento da SQN 7 ^,^ 14 ^,^ 18 . Reed et al., em um estudo feito ao longo de 11 anos com 11 pacientes tratados preferencialmente com transposição de VRE, observaram resolução da hematúria em todos os pacientes e melhora ou resolução da dor em oito deles, sem nenhuma complicação pós-operatória. Relataram também uma taxa de reintervenção de 27% 20 ;
- Autotransplante do rim esquerdo: procedimento altamente invasivo que envolve nefrectomia e transplante renal para a fossa ilíaca 3 . É considerado um procedimento completo por ser eficaz na normalização dos níveis pressóricos da VRE e por corrigir uma possível ptose renal posterior, sendo associado a excelentes resultados e baixa morbidade 3 ^,^ 7 ^,^ 21 . Entretanto, riscos adicionais devem ser levados em consideração, como tempo de isquemia renal, anastomoses de artéria renal e ureter, e necessidade de grande exposição cirúrgica 3 ^,^ 7 ;
- Transposição da AMS: a transposição da AMS de sua origem aórtica para ser reimplantada em um ponto inferior à VRE, procedimento mais complexo de ser realizado se comparado à transposição venosa, é uma alternativa cirúrgica que possui pouca exposição retroperitoneal e reduz o risco de trombose da VRE 3 ^,^ 7 ^,^ 16 . Entretanto, o risco significante de trombose arterial – devido à reanastomose – e isquemia mesentérica, juntamente à elevada taxa de complicações pós-operatórias, tornam seu uso raro 3 ^,^ 7 ^,^ 16 ^,^ 18 ;
- Nefropexia: modalidade terapêutica descrita inicialmente na década de 1980 16 . A nefropexia simples com excisão das varicosidades, um procedimento resolutivo apenas da ptose renal, tem sido desencorajada por não tratar a fisiopatologia primária 21 ^,^ 22 . De acordo com Hmida et al., a nefropexia é usada para o tratamento etiológico quando ocorre concomitante com o abaixamento da VRE através de lobectomia e abordagem retroperitoneal. Tal procedimento, por apresentar baixo risco de lesão de órgãos intraperitoneais, pode ser encarado como uma opção para o tratamento de pacientes jovens 22 ;
- Nefrectomia: é o mais radical dos procedimentos cirúrgicos, recomendado em casos de persistência da hematúria após diferentes formas de abordagens terapêuticas, especialmente após a transposição da VRE 7 ;
- Derivação renocaval: técnica que utiliza a veia safena magna como derivação sem a necessidade de transposição da VRE 17 . A veia safena é anastomosada proximalmente na VCI, abaixo da VRE, e distalmente na VRE. Ambas as anastomoses são realizadas com clampeamento parcial para que interfiram pouco na hemodinâmica venosa. As veias lombares, a veia gonadal e a veia adrenal esquerda não necessitam ser ligadas se não apresentarem refluxo, pois não afetam as anastomoses. Trata-se de um procedimento com resultados em longo prazo e experiência limitados 3 ;
- Transposição da VGE: procedimento no qual a VGE é exposta via mesocolotransverso, isolada, transeccionada distalmente e reimplantada na VCI abaixo dos vasos mesentéricos inferiores com sutura interrompida com fio 6.0 de polipropileno 3 ^,^ 17 .

As técnicas laparoscópicas têm ganhado destaque com o avanço da tecnologia em cirurgia minimamente invasiva 18 . A nefrectomia e o autotransplante laparoscópicos evitam extensas incisões abdominais, estando associados a menor morbidade e desconforto pós-operatórios, com tempo de estadia hospitalar reduzido 16 . Procedimentos de derivação esplenorrenal e transposição de VRE por laparoscopia também foram relatados. Ambas as técnicas registraram melhoras nos sintomas de dor dos pacientes; entretanto, na técnica esplenorrenal, há risco de se causar complicações ao baço e de a hipertensão da VRE permanecer 7 ^,^ 18 . Thaveau et al. 23 , em 2015, reportaram o uso de sistemas robotizados para transposição da VRE seguida de embolização de veia ovariana, de modo que a paciente, seis meses após a operação, encontrava-se assintomática. 

#### Cirurgia aberta para SQN posterior

 A VRE retroaórtica, comprimida entre a coluna vertebral e a aorta abdominal, produz a mesma sintomatologia que a variante anterior da síndrome 17 . A cirurgia aberta com transposição anterior da VRE tem sido o tratamento recomendado para SQN posterior 24 . O procedimento consiste na excisão da VRE com pequena margem da parede da VCI, translocação da veia para uma posição anterior à aorta e sua reimplantação na VCI – geralmente em posição superior à que se encontrava anteriormente, de forma a livrar a pressão 17 . Deser et al. 25 relataram o uso bem-sucedido de prótese de politetrafluoretileno (PTFE) para *bypass* renocaval em paciente com *nutcracker*. Entretanto, o PTFE não é recomendado como opção primária para esse procedimento devido ao risco precoce de trombose e infecção 3 . 

### Tratamento endovascular

 A cirurgia endovascular tem sido uma forma de intervenção cada vez mais popular entre os especialistas para o tratamento de lesões vasculares, incluindo a SQN 13 ^,^ 14 ^,^ 26 ^,^ 27 . Mesmo que muitos cirurgiões ainda optem pelo método tradicional de cirurgia aberta, esse tipo de intervenção apresenta um risco maior de morbidade e de complicações se comparado com abordagens menos invasivas 13 . Dessa forma, com os casos relatados do uso de terapia endovascular conquistando resultados satisfatórios no tratamento de doenças de obstrução venosa, tal abordagem vem sendo cada vez mais indicada pelos pesquisadores 26 . Um dos maiores estudos realizados com esse tipo de técnica até o momento foi feito comparando 15 pacientes tratados por método endovascular com 5 pacientes tratados por meio de cirurgia aberta. Ele mostrou que, nos anos seguintes aos procedimentos, todos os pacientes tratados com stent estavam assintomáticos, apesar de dois demonstrarem hematúria microscópica persistente após exercício físico e um stent ter migrado para o átrio direito, necessitando de cirurgia 28 ^,^ 29 . 

 No mínimo, 150 casos bem-sucedidos de tratamento endovascular já foram relatados na literatura médica 27 ; contudo, informações sobre o acompanhamento por longo período ainda estão em falta, justificando o receio de se utilizar essa abordagem em pacientes jovens 8 ^,^ 30 ^,^ 31 . Conquanto seja uma opção mais simples e atraente, suas complicações incluem migração do stent, fraturas e oclusão venosa 28 . As técnicas utilizadas são a embolização da VGE e a colocação de stent 15 . 

#### Stent

 A colocação de stent endovascular é uma opção alternativa de tratamento. É usualmente preferível à cirurgia aberta devido ao longo período de congestão renal, por conta da maior possibilidade de complicações nesses casos, e à necessidade de extensa dissecção presentes nesse tipo de operação 6 . 

 O primeiro relato de uso de endopróteses vasculares para o tratamento da SQN data da década de 1990 13 ^,^ 14 ^,^ 18 ^,^ 28 . Nessa época, o Wallstent® foi utilizado, e tal equipamento ainda permanece como o de maior preferência pelos cirurgiões; desde então, muitos outros modelos foram aplicados com bons resultados, como Z-stent® espiral, Nikki® stent, Palmaz® stent e SmartControl® stent 13 ^,^ 32 . Duas das principais pesquisas realizadas com esse tema, ambas de autoria chinesa, reiteram o sucesso do método endovascular. Em 2011, Chen et al. acompanharam 61 pacientes durante 5 anos e 6 meses, obtendo quadro de melhora excelente ou boa para cerca de 59 pacientes, com resolução total ou melhora dos sintomas de dor nos flancos, hematúria e proteinúria 32 . Wang et al. 26 , em 2012, analisaram 30 pacientes, 29 dos quais obtiveram redução dos gradientes de pressão renocaval, calculados comparativamente antes e após os procedimentos, com regressão das queixas de dor, hematúria e varicocele no lado esquerdo em pacientes masculinos em um período de até 6 meses após a colocação do stent. A compressão da VRE é aliviada com o stent na maior parte dos pacientes, embora não se saiba se a queda da pressão renocaval ocorra de maneira imediata ou gradual 32 . 

 O stent ideal deve ter alta força radial para abolir a estenose, boa conformabilidade para se adaptar ao epitélio do vaso e pouco encolhimento no comprimento para permitir um posicionamento adequado 13 ^,^ 26 ^,^ 32 . O SmartControl® stent combina alta força radial e flexibilidade com menos de 8% de encolhimento de seu comprimento. O Palmaz® stent e o Wallstent® têm maior contração do aparelho, que chega a ser de 5 a 25% e de mais de 30%, respectivamente 32 . Os stents autoexpansíveis (Wallstent®) são mais frequentemente utilizados, e a recomendação é de que seja usado um dispositivo de 6 ou 8 centímetros de comprimento, posicionando-o na primeira divisão da VRE 26 . Além disso, para evitar a migração do aparelho, sugere-se que o stent seja cerca de 20% maior que o diâmetro venoso no hilo renal. O tamanho considerado base é de 16 mm de diâmetro por 60 mm de comprimento em pacientes de ascendência ocidental e 14 mm de diâmetro por 60 mm de comprimento em pacientes de ascendência oriental, por conta das menores proporções anatômicas características dessa população 32 . O uso do cateter balão de angioplastia é controverso e não essencial 13 ^,^ 26 ^,^ 32 . Chen et al. recomendam que a expansão do balão somente seja utilizada nos casos em que a estenose da VRE permaneça mesmo após a implantação do stent 32 . 

 Faz-se necessário que os pacientes utilizem medicamento anticoagulante e antiagregante plaquetário durante 2 a 3 meses, período necessário para que ocorra a endotelização completa do stent 19 ^,^ 33 . O regime terapêutico recomendado consiste em 3 dias de heparina de baixo peso molecular, 30 dias de clopidogrel e 3 meses de aspirina 33 . 

 As principais complicações desse meio de intervenção incluem colocação incorreta do stent, migração do dispositivo, deslocamento parcial do stent para a VCI e migração para a região do hilo renal da VRE 7 . Complicações menos comuns incluem embolização do stent, reestenose intra-stent e trombose ou fratura resultando em oclusão venosa 7 ^,^ 19 ^,^ 32 . Essas complicações se devem principalmente ao tipo e tamanho do dispositivo, dilatação do balão e escasso conhecimento do cirurgião que realizou o procedimento 7 ^,^ 34 . 

 Mesmo que a literatura atual sugira que esse procedimento seja seguro, é necessário cautela, pois a taxa de migração do stent é de 7,3% em todos os casos já relatados. A razão da migração pode ser o efeito do movimento cardíaco, atividades muito precoces após a cirurgia, incompatibilidade entre o diâmetro da VRE e o diâmetro do stent, ou posicionamento incorreto 29 . A primeira e mais segura escolha para o tratamento do stent migrado é a remoção percutânea 32 . Entretanto, sob certas circunstâncias, como migração para o coração, uso de stent especial ou endotelização em local inadequado, a remoção percutânea pode ser difícil ou impossível, com necessidade de intervenção cirúrgica, procedimento que possui altas taxas de morbidade 32 ^,^ 35 . 

 Uma forma de stent pouco presente na literatura é o extravascular. Comparado ao stent endovascular, é uma modalidade menos invasiva, se for levada em consideração a possibilidade de migração do dispositivo. Especialmente quando os pacientes são crianças e adolescentes, o stent intravascular deve ser recomendado com cautela, pois a luz da VRE pode aumentar de diâmetro e o aparelho não servir mais durante o desenvolvimento físico. A modalidade extravascular é uma forma alternativa de manter o dispositivo no seu devido lugar 29 . 

#### Embolização da VGE

 Uma vez que a SQN se instala, uma de suas possíveis consequências é a drenagem insuficiente do volume sanguíneo da VGE, ocorrendo acúmulo de sangue e dilatação de suas paredes, podendo resultar em seu rompimento 8 . Pacientes que apresentam essa síndrome, com ou sem hematúria, podem revelar sintomas de insuficiência da veia gonadal, que se apresenta como varicocele no homem e síndrome de congestão pélvica na mulher. Mesmo que o tratamento da estenose da VRE possa aliviar o refluxo gonadal, geralmente não é eficiente em melhorar os sintomas 3 . Para isso, a embolização endovascular das veias gonadais pode resultar em alívio dos sintomas em 56 a 98% dos pacientes. Em casos raros, as complicações incluem o deslocamento das molas (*coils*) para o pulmão 8 . 

 Desde sua introdução em 1993, a embolização transcateter da VGE se tornou o padrão-ouro no tratamento de síndrome da congestão pélvica secundária à insuficiência de veias ovarianas e pélvicas. Diversos agentes “embolizadores” foram descritos, como espuma esclerosante, cola, *plugs* vasculares e molas 36 . 

 O procedimento é tipicamente feito via acesso femoral, embora o acesso jugular também possa ser utilizado 3 . Exames de imagem da VRE devem ser realizados para garantir que a drenagem do rim esquerdo esteja sendo feita, pois pacientes com compressão severa podem ser bastante dependentes do fluxo pélvico 3 ^,^ 37 . 

 A partir da literatura atual, a decisão quanto a tratar uma ou ambas as veias ovarianas deve depender da gravidade dos sintomas, do grau de refluxo em cada veia ovariana e da anatomia das varicosidades pélvicas. A análise clínica e a experiência devem ser utilizadas para guiar a emboloterapia em conjunto com sintomas, anatomia e estudos funcionais 36 . 

## CONCLUSÃO

 Apesar de rara, a SQN está presente na prática médica. Faz-se necessário, portanto, o seu conhecimento para a realização de correto diagnóstico e manejo do paciente. Os achados clínicos e a alta suspeição diagnóstica são fatores decisivos para o estabelecimento da conduta, especialmente em pacientes que não realizaram exames de imagem previamente. As opções cirúrgicas são amplas e apenas o domínio aprofundado teórico-prático dessas técnicas permite a escolha correta diante dos diferentes tipos de pacientes portadores da síndrome. Sendo assim, torna-se evidente a necessidade de fortalecer os conhecimentos teóricos embasados em SQN, tal como realizado nesta revisão. 

## REFERÊNCIAS
